# Mesoscale Numerical Analysis of Fiber-Reinforced Sand with Different Fiber Orientations Subjected to Seepage-Induced Erosion Based on DEM

**DOI:** 10.3390/ma16010335

**Published:** 2022-12-29

**Authors:** Shengtao Yang, Yan Lv, Yuanyuan He, Minggang Pang, Xiaozhen Ma

**Affiliations:** College of Construction Engineering, Jilin University, Changchun 130026, China

**Keywords:** seepage-induced erosion, fiber-reinforced sand, DEM-darcy coupling, fiber orientation, mesoscopic mechanism

## Abstract

This paper focuses on the effect of fiber orientation on the resistance of seepage-induced erosion in fiber-reinforced sand. To clarify the discrepancy and mechanism of different-oriented fibers improving the resistance of the sand matrix, a series of DEM-Darcy coupling simulations were conducted. The microscopic parameters of fiber-reinforced sand were confirmed by the rigorous calibration procedure. The fibers perpendicular to the seepage direction were found to increase the difficulty of moving fluid through the specimen and significantly reduce the erosion rate of the specimen. These macroscopic behaviors acquired corresponding explanations at the mesoscopic scale, including the evolution of fiber-sand contact orientation, coordination number, average normal contact force, tensile force, and energy dissipation. According to the simulation results, it is found that the highest proportion of tensile force in perpendicular fibers can reach 80%, while the parallel fibers are only 40%, which indicates that the perpendicular fibers have a significant netting effect. The mesoscopic behaviors reasonably revealed the role of the fibers with different orientations on the sand matrix during the seepage. This study is beneficial for further understanding the mechanical behaviors of fiber-reinforced sand under seepage-induced erosion in safety engineering.

## 1. Introduction

In geotechnical engineering, it is found that the strength of a sand or soil matrix can be enhanced by the addition of tensile materials. Compared with other reinforcement materials, fiber materials are widely used because of their high strength, easy dispersion, and stability [1]. As a non-rigid and non-brittle material, fiber-reinforced soil (FRS) is an excellent material used in slope [2,3], retaining wall [4], embankment, and road base fill reinforcement projects [5]. However, the seepage erosion of the FRS is unavoidable in these engineering applications. The forced migration of soil particles driven by seepage force can threaten the structural integrity and stability of embankment foundations and slopes. Laboratory mechanical tests on fiber-reinforced sand have demonstrated that both natural [6,7] and synthetic [8] fibers can perform a reinforcing effect in the specimen, thereby increasing the strength of soil/sand. Nonetheless, the behavior of fibers during the seepage erosion can be different from the triaxial compression test or tamping test because the specimen tends to be looser with the process of seepage erosion while denser under mechanical tests. Thus, it is desirable to investigate the role of fibers in the fiber-reinforced sand during seepage-induced erosion. Clarifying the mechanical behavior of FRS suffered by the scour of the flow will help to mitigate seepage-induced adverse effects and enhance the stability of the fiber-soil mixtures.

The previous experimental works about the scouring resistance of fiber-reinforced soils have found that the resistance to erosion by the water flow of a sand matrix can be greatly enhanced with the addition of fibers [9,10]. Meanwhile, the seepage velocity of FRS is decreased in comparison with the pure sand matrix. Estabragh et al. [11] experimentally tested the difference between seepage velocity and piping resistance for reinforced and unreinforced silty sand specimens. Das et al. respectively investigated the influence of randomly added fibers on the piping behavior of fly ash [12] and silty sand (containing fines) [13]. These findings all pointed to the fact that the fibers play a reinforcing role when the fiber-reinforced soils are subjected to seepage flow. Yang et al. [14] pointed out that the fiber netting effect and the vertical reinforcing effect were the main mechanisms that can effectively improve erosion resistance. Furumoto et al. [15] reported that the presence of fibers effectively restricted the movement of soil particles in the permeability tests. The fiber characteristics that affect erosion resistance and seepage of fiber-reinforced soils were studied mostly in terms of the factors of fiber length and content [13,16,17,18]. However, the influence of fiber orientation on erosion resistance has been little discussed. Yang, Wei, Adilehou, and Ho [14] indicated that the internal erosion resistance of fiber-reinforced soil was not significantly improved during seepage-induced erosion when the fiber orientation was parallel to the seepage direction.

The discrete element method (DEM) is an effective approach to investigating the mechanical behavior of geomaterials at the particle scale. Recently, many scholars have employed the DEM to analyze the mechanical mechanisms of fiber-reinforced soil from a mesoscopic perspective. The previous works based on the DEM about fiber-reinforced soil simulate the tamping test [19], direct shear tests [20], and biaxial [21] or triaxial [22] compressive tests by constructing the soil-fiber discrete element system. These studies provide the concept for constructing discrete element models of fiber-reinforced soils: a system of strips of fibers (modeled by numerous continuously arranged balls bonded together) and spherical soil particles. The DEM modeling of a non-binary mixture subjected to seepage erosion has been performed in previous simulations. Zhang et al. [23] investigated the stress-strain behavior of gap-graded soils after internal erosion based on the DEM. Gu, et al. [24] developed the predictive model of clayed sand during seepage-induced erosion by coupling the DEM and CFD (computational fluid dynamics). Xiao and Wang [25] simulated and analyzed the movement characteristics of fine particles around the sheet piles/cutoff wall during seepage. These studies provide approaches and schemes to simulate geomaterials in seepage-induced scour.

For the study of DEM-Darcy coupling, Tang et al. [26] employed the coupling model of DEM-Darcy based on the particle flow code (PFC) version 6.0 software platform to predict the erosion rate of sand and the shape of erosion pores, which has demonstrated the effectiveness of the coupling model for simulating the sand erosion. Wang et al. [27] used the DEM-Darcy model to explore the different influencing factors (gap ratio range and fines content range) in seepage-induced erosion of bimodal soils. The exchange of computational information between DEM and Darcy’s module was implemented by using the *FiPy* solver to process the fluid data. *FiPy* was filed as belonging to the Python library, which has been built in PFC3D. However, these simulations have rarely involved the discussion of variations in the mesostructure. Furthermore, the DEM simulations of fiber-reinforced soil have not been widely conducted on scour resistance or seepage research. There is a lack of discussion on the influence of fiber orientation on the stability of fiber-reinforced soils during water scouring. Besides, experimental studies can only obtain the macroscopic behavior, and the reinforcement mechanism of fibers through the microscopic behavior of fiber-soil mixtures deserves to be revealed. These aspects are the motivation for the present study. To fill the gap in terms of the effects of fiber orientations on fiber-reinforced sands during seepage-induced erosion, the main purpose of this article is to discuss the macroscopic observation of specimens with different orientated distribution fibers and the variation of the fluid field under seepage-induced erosion, and the discrepancy of the macroscopic behavior was revealed from a mesoscopic viewpoint. First, fiber-reinforced sand specimens with different orientated fibers and fluid fields matched to the specimen size were constructed. Then, the microscopic parameters of fiber-reinforced sand were calibrated by the direct shear tests. For the variation of the fluid field, the difference in the evolution of fluid cells’ porosity, permeability, water pressure, and drag force was studied. For the fiber-reinforced sand, macroscopically, the difference in erosion ratio between specimens with different fiber orientations was presented. Microscopically, the particle-displacement field, evolution of fiber-sand contact behavior, alternation of force inside fibers, and dissipation of system energy were analyzed. This research provided a typical study on the two-way coupling of fluid-solid behavior in DEM simulations. These studies can contribute to a better understanding of the role of fibers in enhancing scour resistance of sand matrix.

## 2. DEM Simulation of Seepage-Induced Erosion

### 2.1. DEM-Darcy Coupling Progress

In the current study, discrete modeling of fiber-reinforced sand is constructed using a particle flow code in three-dimensional (PFC3D) version 6.0 software developed by Itasca International Consulting Group, Inc (Falcon Heights, MN, USA) [28]. The fluid flow module has been built in PFC3D. However, it can only achieve a one-way coupling of fluid to particle action without the assistance of other programming languages. The two-way coupling of PFC3D can consecutively and concurrently exchange the information between particles and fluid through custom Fish functions and Python script files to solve Darcy’s law and the continuity equation.

**Figure 1:**
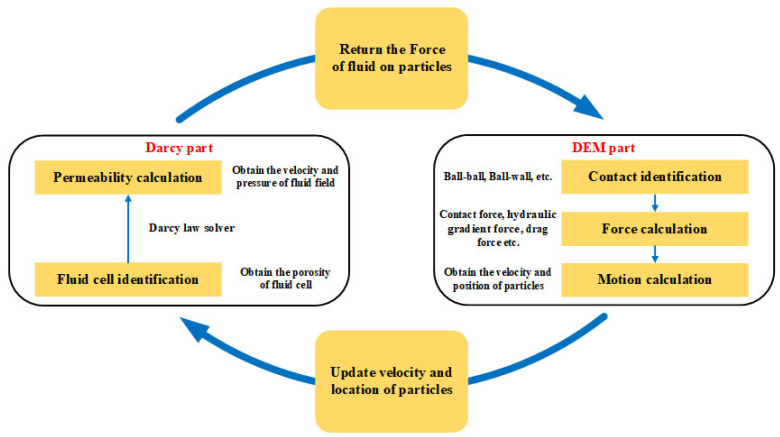
Computational procedure of DEM-Darcy coupling.

The typical procedure of DEM-Darcy coupling is based on the solution of Darcy’s Law in porous media. Figure 1 illustrates the computational procedure of the DEM-Darcy coupling. For the Darcy part, the main purpose is to obtain the parameters (porosity, permeability coefficient, etc.) of the fluid field. The description of Darcy’s law used in the flow of porous media with a low Reynolds number is as follows:

(1) $$\vec{v}=\frac{K}{\mu \varepsilon }\vec{\nabla }p$$

where the $\vec{v}$ and p are the mean velocity and pressure of the fluid, respectively; K and ε are the matrix of the permeability coefficient and porosity of the fluid, respectively; and μ is the fluid viscosity. The matrix permeability coefficient K is estimated according to the Kozeny-Carman equation in PFC3D:

(2) $$K_{\left(\varepsilon \right)}=\left\{\begin{array}{cc} \frac{1}{180}\frac{\varepsilon^{3}}{{\left(1-\varepsilon \right)}^{2}}{\left(2r_{e}\right)}^{2} & \varepsilon \leq 0.7\\ K_{\left(0.7\right)} & \varepsilon >0.7 \end{array}\right.$$

where the $r_{e}$ is the mean radius of particles in the fluid field; and the matrix porosity ε can be monitored in the fluid field, the calculation of ε is as follows:

(3) $$\varepsilon =1-\frac{4\pi }{3V}\sum_{i=1}^{n}r_{i}^{3}$$

where the V is the volume of the whole numerical model and $r_{i}$ is the radius of each particle. Additionally, it was specified that the $K_{\left(\varepsilon \right)}$ is taken as $K_{\left(0.7\right)}$ when the porosity ε of the sample exceeded 0.7. Once the $K_{\left(\varepsilon \right)}$ of the particle system is altered, the velocity and pressure of the fluid field will also be altered. Therefore, the force that fluid elements exert on the particles will be updated as this progress is made.

For the DEM part, the calculation is performed in the DEM by the alternating operation of Newton’s second law applied to the balls and the force-displacement law at the contacts. In addition to the contact forces between the particles (or the particle wall), there is the drag force and hydraulic gradient force generated by the fluid elements. The drag force on the individual particle is defined as:

(4) $$f_{\mathrm{drag}}=\frac{1}{2}C_{d}\rho_{f}\pi r^{2}\left|\vec{u}-\vec{v}\right|(\vec{u}-\vec{v})$$

where the $\rho_{f}$ is the fluid density, and $\vec{u}$ and $\vec{v}$ are the velocity of particle and fluid, respectively. $C_{d}$ is the drag force coefficient, which can be defined as:

(5) $$C_{d}={\left(0.63+\frac{4.8}{\sqrt{Re_{p}}}\right)}^{2}$$

where $Re_{p}$ is the Reynolds number of the single particle, it can be defined as:

(6) $$Re_{p}=\frac{2\rho_{f}r\left|\vec{u}-\vec{v}\right|}{\mu_{}}$$

where the $\mu_{}$ is the fluid viscosity. The hydraulic gradient force is derived from the pressure acting on the particles in the flow with a pressure gradient $\vec{\nabla }p$. The hydraulic gradient force on the individual particle can be express as:

(7) $$f_{\mathrm{pressure}}=\frac{4}{3}\pi r^{3}\vec{\nabla }p$$

The drag force, hydraulic gradient force, and contact force between the particles (or particle and wall) together determine the motion of the particles. The change in velocity and location of particles can alter the distribution of the particles in the fluid field, which in turn affects the porosity and permeability of the particle system in the fluid field. Therefore, the cycle between the Darcy and DEM parts can achieve a two-way coupling of fluid and particles.

### 2.2. DEM Modeling of Fiber-Reinforced Sand

**Figure 2:**
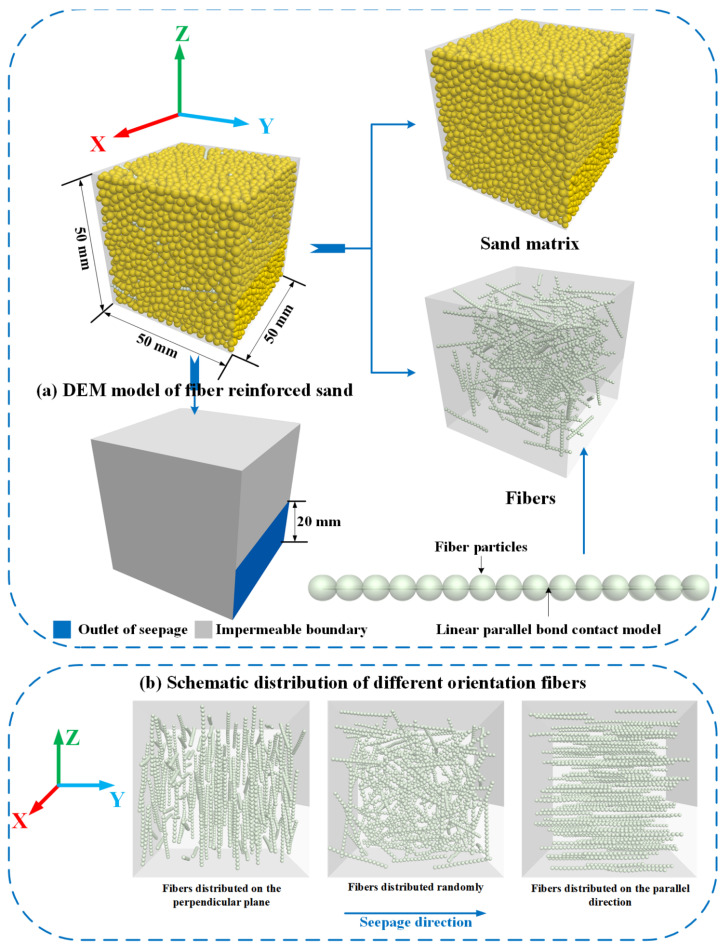
DEM-Darcy (two-way) coupling model of fiber-reinforced sand: (**a**) DEM model of fiber-reinforced sand; (**b**) three distribution states of fibers.

For the simulation of seepage-induced erosion, the size of the fiber-reinforced sand specimen was 50 mm in width (X-axis), 50 mm in length (Y-axis), and 50 mm in height (Z-axis), as shown in Figure 2a. Two walls as the inlet and outlet of seepage and four walls as impermeable boundaries were generated in numerical seepage-induced erosion. To prevent the leakage of particles during the generation, the six walls were extended by a factor of 0.2 on the outside. Sand particles were generated as rigid balls. To improve the computational efficiency of the simulation, the size of the sand particles was scaled up. In the DEM simulation for seepage erosion in gap-graded granular sands/soils, it was discovered that the distribution of particle size, such as coarse particle size distribution and fines content, is an important factor affecting the migration of particles [29,30,31]. For instance, Xiong, Zhang, Sun, Yin, and Chen [30] pointed out that the grain size distribution of coarse grain has a prominent impact on the deep penetration of fines. Qian, Li, Yin, and Yang [31] found that the loss ratio of fines in gap graded granular soils is controlled by the fines content and mean particle size ratio. Hence, to avoid forming a sand matrix as a mixture of coarse and fines caused the effect of particle size distribution. In the present study, the distribution of the radii of the sand particles was chosen to be normal in the range of 1 mm to 1.5 mm, which can generate a homogenous and stable sand matrix. The fiber was modeled as thread-like shapes, and its length was determined by the number of constituent balls. Here, the strips of fibers are modeled by 15 cluster balls with the same radius of 1 mm bonded together, as shown in Figure 2a.

To prepare the DEM model of the mixture of sand matrix and fibers, the wall with the orifice was created to be intact at the beginning. Then, the sand particles and fibers were placed in the material vessel. The fibers were first generated as ‘clumps’ by the custom-specified templates in this study; as a consequence, the distribution of fibers can be manipulated in a prescribed orientation. Additionally, the fiber content can be determined by the generation number of fiber ‘clumps’ in this step, and here the fiber content (gravimetrical) was kept at 1% after calculating. Then, the ‘clump pebble’ elements of fiber ‘clumps’ were replaced by ball elements using customized code. As shown in Figure 2b, the orientation of fibers can be divided into those along the seepage direction and those perpendicular to the seepage direction. Additionally, the specimen with free distribution of fibers was used to compare the effect of fiber orientation.

In the initial phase of the sand particle and fiber placement, the friction coefficient is set to a very low value to reduce the overlap on the particle surfaces to an acceptable level. Once the fiber elements had been replaced, the predefined contact types and calibrated parameters were implemented in the DEM specimen of fiber-reinforced sand. The final phase before performing the seepage simulation was the consolidation of the specimen to ensure the compactness of the interparticle contact. A uniform confining pressure (200 kPa) was applied to the specimens with three fiber orientations by the servo-control mechanism.

### 2.3. Contact Model in DEM Model of the FRS

**Figure 3:**
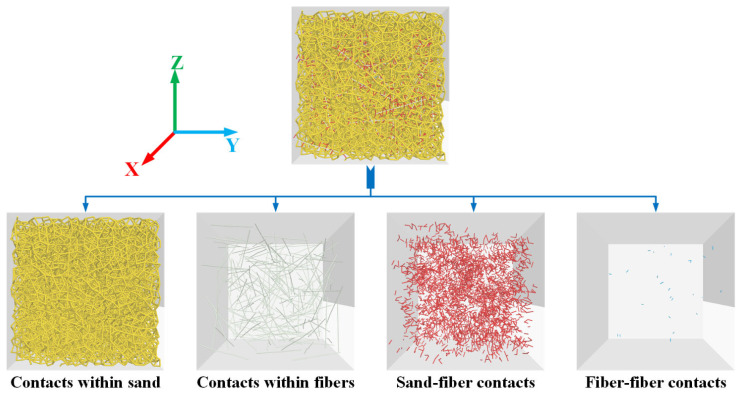
Contact types in the DEM model of fiber-reinforced sand.

For a binary mixture, the selection of the contact model and microscopic parameters needs to be deliberated. There are four types of contact in the fiber-reinforced sand sample: within sand particles, within fibers, fiber-sand particles, and fiber-fiber, as shown in Figure 3. Table 1 displays the contact model corresponding to each contact type of fiber-reinforced sand in this study. For sand particles, the anisotropy of this material cannot be reflected in the simulation because they are modeled as spherical particles to improve the efficiency of the calculation. However, the rotation effect of spherical particles can be reasonably offset to a certain extent if the rolling resistance (RR) linear model is chosen as the contact model within sand particles. In previous DEM simulations, the RR linear model has been used to simulate the interaction between sand particles, such as the sand particles in fiber-reinforced sand [20], sand-rubber mixtures [32], and pure sand [33]. Therefore, the RR linear model was selected to capture the contact behavior of sand-sand in this study. The strips of fibers are modeled as multiple continuously aligned spherical particles aggregated together by a bond contact model. There are two main bond contact models built in PFC3D: the linear parallel bond (LPB) model and the linear contact bond (LCB) model. The tangential force existed in fibers owing to the extrusion of sand matrix. The LPB model employed between discrete elements can withstand tangential forces owing to parallel bonds. Tuan [34] pointed out that the LPB model is more suitable to model the firm and touch fiber. Therefore, the LPB model was selected to capture the shearing behavior as well as the tension and compression behaviors. The linear model was selected to simulate the mechanical behavior of sand-fiber contacts and fiber-fiber contacts.

### 2.4. Calibration of Sand-Fiber Mixture Parameters

The microscopic parameters of fiber-reinforced sand, including the contact between the discrete elements (sand and fiber), were difficult to measure throughout the seepage tests in the laboratory. Nevertheless, Knapen et al. [35] pointed out that the shear strength seems to be the most appropriate parameter for representing the erosion resistance of the soil matrix during concentrated flow. Furthermore, Gu, Huang, Liu, Zhang, and Gao [24] performed a coupled CFD-DEM simulation of seepage-induced erosion in clay sand based on the microscopic parameters calibrated by direct shear tests. Previous DEM studies have confirmed that the macroscopic behavior of FRS can be reasonably captured by matching the microscale parameters obtained from the data of direct shear tests and triaxial compression tests [20,22]. Therefore, the microscopic parameters of FRS were accepted by the laboratory results of direct shear tests in this study.

The numerical specimen of fiber-reinforced sand was established with reference to the materials reported by Darvishi and Erken [36]. Gong, Nie, and Xu [20] have carried out the parameter calibration and subsequent DEM simulation of the direct shear test based on Darvishi and Erken’s experimental data. The setting of DEM parameters in the present study is also based on the published experimental results of Darvishi and Erken [36]. We recalibrated the DEM parameters of fiber-reinforced sand using the shear stress-horizontal displacement curves with fiber content (1.0% here) and sand particle diameter distribution (according to the experimental grain size distribution here) different from the calibration progress of Gong, Nie, and Xu [20]. Now, the primary fundamental parameters of the elements (sand particles and fibers) that comprise the specimen used for calibration are introduced below.

**Figure 4:**
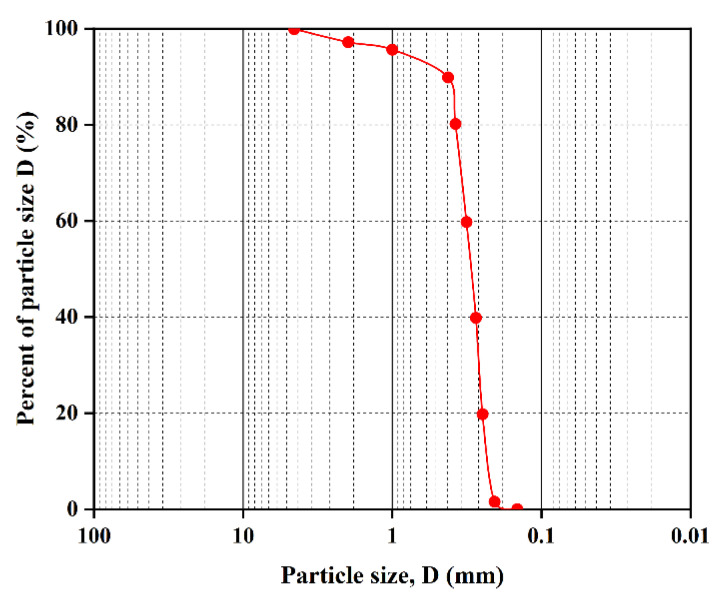
Grain size distribution of the sand (this distribution was only used to calibrate parameters and not for subsequent seepage simulations).

The specific gravity of sand particles was 2.69 g/cm^3^. The material of the fiber was polypropylene, and the specific gravity was 0.91 g/cm^3^. The range of porosity of the sand matrix was 0.353–0.467. The size distribution of the diameter of sand particles in the laboratory was given, as shown in Figure 4. Gong assigned a normal distribution from 1.0 to 1.4 mm for sand particle radius in the DEM simulation, but this was not according to the gradation curve. In this calibration, the distribution of the sand matrix diameter was chosen in accordance with the gradation curves and scaled up to decrease the high simulation costs. The porosity of the sand matrix was 0.4. The actual length of the fibers was 15 mm, and the tensile strength of the fibers was 570–660 MPa. In the DEM simulation, the length of the fibers was 15 mm, and the slenderness ratio of the fiber was dependent on the number of bonded series of fiber particles. The fiber content of the fiber-reinforced sand was selected at 1.0%, which was the same as the laboratory preparation.

**Figure 5:**
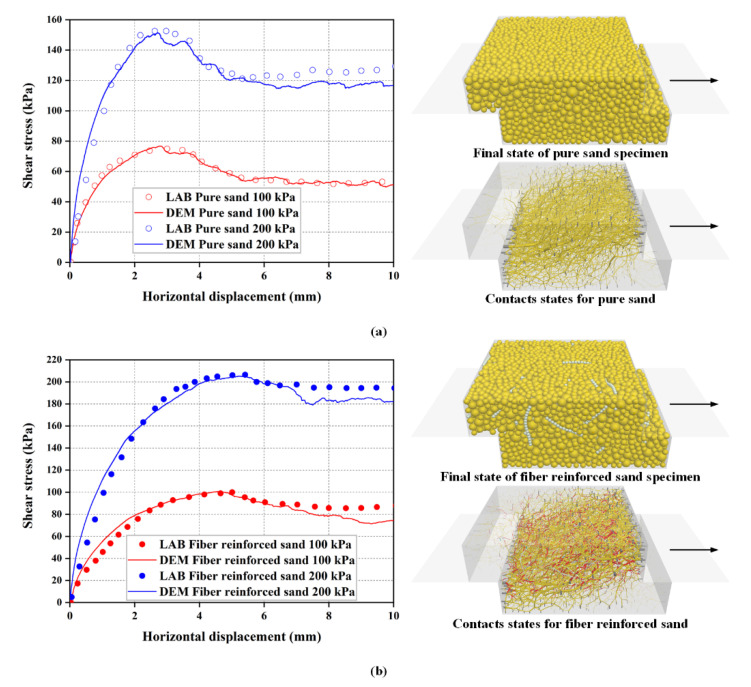
Calibration of microscopic parameters: (**a**) comparison of the direct shear testing result between laboratory and simulation for pure sand; (**b**) comparison of the direct shear testing result between laboratory and simulation for fiber-reinforced sand.

The basic sequence of calibration is to first determine the parameters of pure sand (0%), then the fiber parameters are calibrated through the shear stress-horizontal curves of the sand-fiber mixture based on the known sand parameters for the final calibration. Figure 5 presents the comparison of the direct shear testing result under the normal pressure of 100 kPa and 200 kPa between the laboratory and simulation for pure sand and fiber-reinforced sand. The predicted DEM responses agree well with the laboratory data, thus indicating that the mechanical behavior of fiber-sand composites can be captured by the proposed DEM model. Table 2 shows the microscopic non-bonding parameters calibrated in this study. Table 3 shows the bonding parameters of fiber internal particles. Note that the bond strength was chosen at a larger value of 1 × 10^12^ Pa to prevent breakage inside individual fibers.

### 2.5. Implementation of a Numerical Seepage-Induced Erosion System

**Figure 6:**
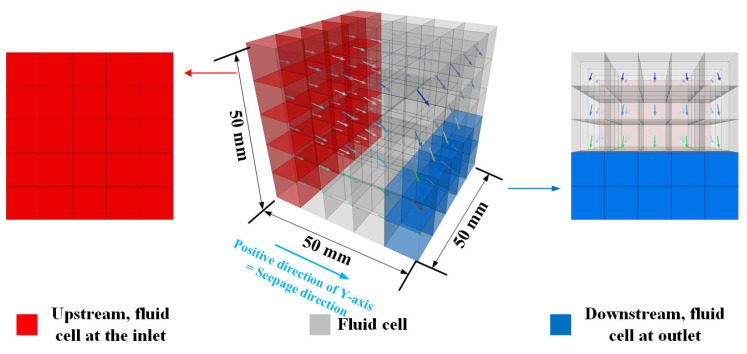
Implementation of the fluid field for two-way coupling (arrows denote the flow direction of each fluid cell).

The seepage-induced erosion system implemented in PFC3D is shown in Figure 6. The applied numerical seepage erosion strategy is referred to in the similar simulations conducted by Wang, Huang, Tang, Hu and Ling [26,27]. Differently, the horizontal seepage (along the positive direction of the Y-axis in this simulation) is employed to better obtain the infiltration path. A pressure difference is applied by presetting a certain pressure on the fluid cell at the inlet and outlet to form a hydraulic gradient of 1. Then, the seepage can be induced along the positive direction of the Y-axis by the hydraulic gradient. Note that the outlet of the fluid field is overlapped with the orifice in the boundary to ensure the flushed sand particles and fibers can flow out of the orifice. At a distance of 20 mm (along the Y-axis) from the outlet, which is considered to be the terminal of total particles outside the fluid field and the particles. The particles moved there by the remaining dynamics and collisions are finally destroyed.

The division of the fluid mesh is important for the motion analysis of the fluid. According to the suggestions of Wang, Huang, Tang, Hu, and Ling [27] and Itasca [28], the size of the fluid mesh should exceed 1-4 times the average diameter of particles in the domain of individual fluid mesh, while meeting the requirement of at least five meshes in the X, Y, and Z directions to accurately capture the flow state of the fluid. Therefore, the X, Y, and Z directions are all divided into five fluid cells in the fluid field with a size of 50 mm (X) × 50 mm (Y) × 50 mm (Z).

During the seepage-induced erosion, the density and viscosity of the fluid were set at 1 × 10^3^ kg/m^3^ and 1 × 10^−3^ Pa·s, respectively. The gravity of the DEM system was set to zero to ensure the sand particles in the fluid field are isotropic.

Once the setup of the fluid field is established, the particles downstream are progressively washed out, and the particles upstream constantly move towards the outlet to replenish the number of particles there. Overall, the number of particles in the upstream area keeps decreasing, while the number of particles in the downstream area initially remains relatively variable before finally decreasing. Meanwhile, the fluid field is also changed owing to the evolution of the number of particles within it, and these changes can be monitored in each fluid cell.

## 3. Results of the Two-Way Coupling Simulation

### 3.1. Variation of Fluid Field

#### 3.1.1. Measured Fluid Cell Porosity

The particles of the numerical specimen are continuously flowing towards the orifice under the action of fluid, which can be migrated along the direction of the fluid flow during the seepage-induced erosion. The porosity of each fluid cell can be monitored by calculating the volume of the particles inside each fluid cell and the volume of each fluid cell; the porosity P of each fluid cell can be defined as:

(8) $$P=\frac{V_{\mathrm{cell}}-V_{\mathrm{partcles}}}{V_{\mathrm{cell}}}$$

where the $V_{\mathrm{partcles}}$ is the total volume of sand particles inside the fluid cell and $V_{\mathrm{cell}}$ is the volume of the fluid cell.

**Figure 7:**
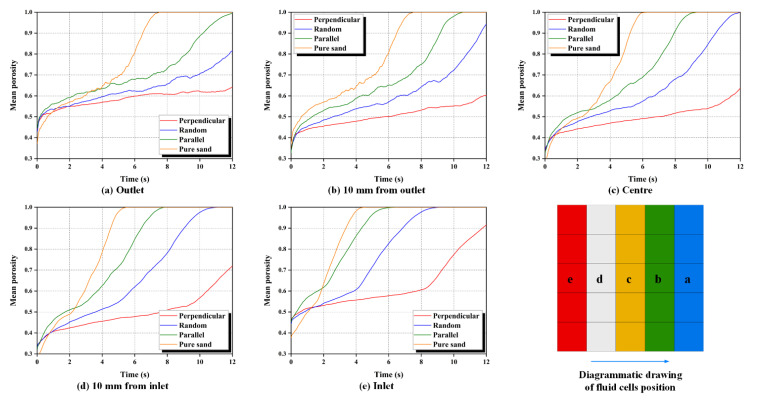
Evolution of the mean porosity of fluid cells.

Figure 7 presents the evolution of the mean porosity of fluid cells at different locations along the seepage direction for pure sand and fiber-reinforced sands. The mean porosity of the fluid cells at the inlet is the first to reach one, and the fluid cells at the outlet are the last to reach 1. Meanwhile, it can be seen that pure sand has a relatively higher ascending rate of mean porosity at each location compared to fiber-reinforced sand. This indicates that the presence of fibers provides reinforcement and support for the sand matrix, which hinders the sand particles from being washed. The fibers distributed on the surface perpendicular to the seepage direction perform the optimal hindrance effect.

#### 3.1.2. Water Pressure and Permeability

**Figure 8:**
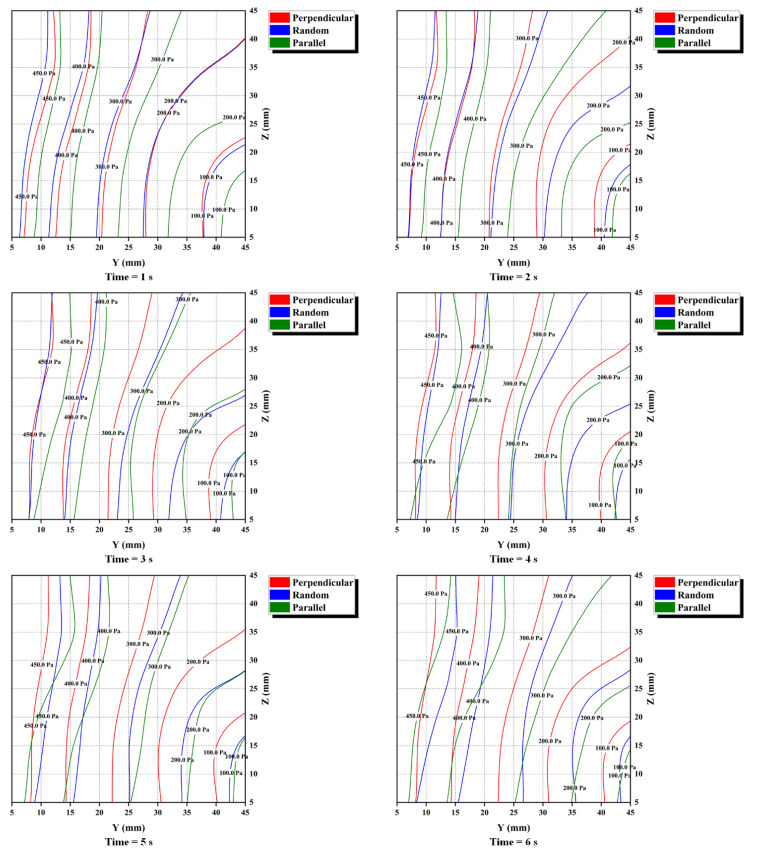
Contour of the evolution of fluid element pressure in the YZ plane with time.

To analyze the pressure field of fluid cells during the seepage-induced erosion for different orientation specimens, the pressure values of the fluid cells at different moments were extracted to plot the piezometric contour of fluid. Figure 8 shows the piezometric contour plots of the fluid element in the Y-Z plane at the moments of 1 s, 2 s, 3 s, 4 s, 5 s, and 6 s (since the start of seepage), respectively. The piezometric contour plots after 6 s are not shown because the difference in fluid pressure between the three fiber orientation specimens is not significant since then. As a whole, the pressure of each fluid cell is increased with the loss of particles. Therefore, the contour of the fluid element pressure will progressively converge towards the outlet. In addition, the pressure contours of the three specimens in order of being away from the outlet are parallel, random, and perpendicular for each moment. Following Darcy’s law, a larger hydraulic gradient is needed for a given water flow as the permeability of the specimen decreases. Liu et al. [37] have shown that the negative pore water pressure increases with the root volume ratio under the drying condition (roots are unable to uptake water and perform similarly to the fibers in the sand matrix). This conclusion demonstrates that the presence of strips of reinforcement (such as fibers and roots that cannot uptake water) in the soil/sand matrix can decrease the water permeability of the matrix and increase the water pressure. In conjunction with the evolution of piezometric contour plots (Figure 8), these mean that the specimen with perpendicular to seepage direction fibers has the lowest water permeability, followed by random and parallel to seepage direction specimens.

**Figure 9:**
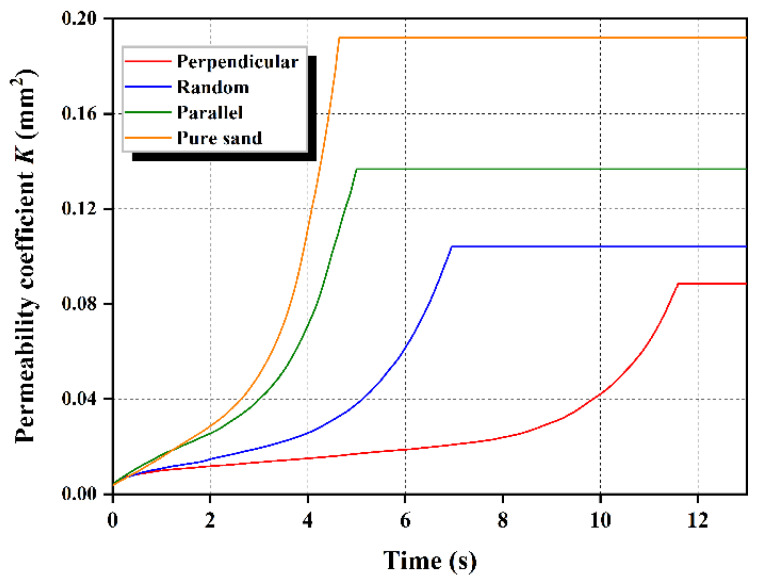
Permeability variation of pure sand and fiber-reinforced sand with seepage-induced erosion.

The permeability coefficient K can be calculated according to Equation (2), the ε in Equation (2) is defined as the average value of the porosity of each fluid cell in this study. The calculation of K was conducted until the measured average porosity value reached 0.7. Figure 9 illustrates the permeability coefficients of pure sand and fiber-reinforced sands during seepage-induced erosion. This also confirms that the permeability of the fiber-reinforced sand is lowest with the perpendicularly orientated fibers, followed by those with random orientation and, finally, those with parallel orientation.

#### 3.1.3. Drag Force

**Figure 10:**
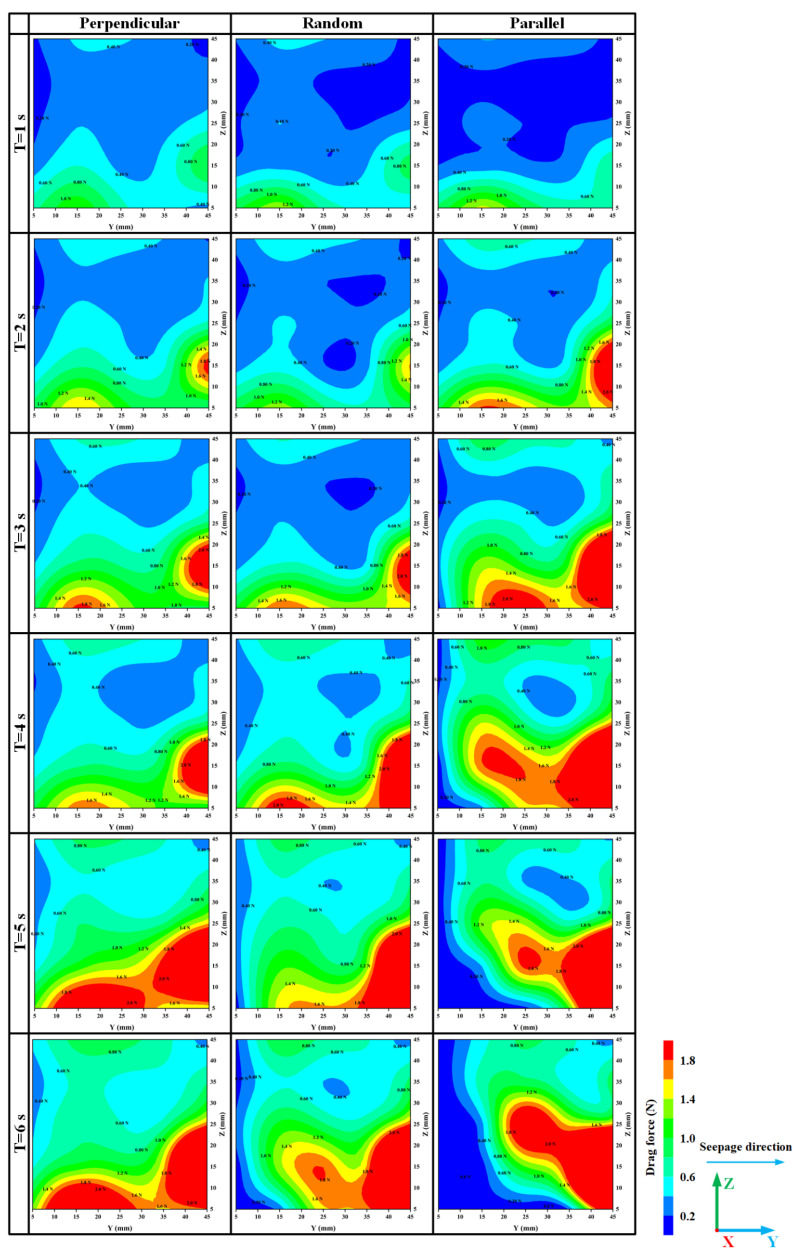
Evolution of the distribution of drag force in the vessel YZ plane with time.

The drag force of the fluid on the particles can be monitored and recorded in each fluid cell. The drag force is the major contributor to the total interaction force between the two phases [38]. Guo and Yu [39] reported that the region distributed with a larger drag force was the first to be eroded due to higher fluid flow. This indicates that the magnitude of the drag force is related to the fluid flow. Figure 10 illustrates the contour graph of the drag force on the specimens with different oriented fibers at the moments of 1 s, 2 s, 3 s, 4 s, 5 s, and 6 s (since the start of seepage), respectively. Over time, it can be observed that the drag force near the outlet increases the fastest in the specimen with parallel seepage direction fibers. This observation indicates that erosion near the outlet first appears in the parallel fiber specimen, followed by random fibers, and lastly perpendicular fibers. In addition, because of the faster rate of particle loss, the distribution areas of the 0 value of drag force near the inlet spread fastest in parallel fibers specimens as well, as shown in Figure 10 at the time of 5 s and 6 s. At moments 5 s and 6 s, the perpendicular fiber specimen shows the maximum area of high drag force distribution. Since the hydraulic gradient between the outlet and inlet is constant, a drag force distribution area with a high value formed in the perpendicularly distributed fiber specimen near the outlet indicates that there are a higher number of remaining particles in the specimen with perpendicularly distributed fibers. Meanwhile, the distribution of drag force with a high value for the parallel specimen is the smallest at the time of 5 s and 6 s, which indicates that the remaining particles in the parallel specimen are the least compared to other specimens. Altogether, the evolution of drag force contour plots for specimen with perpendicular fibers is lagging behind that of specimens with parallel fibers, while that of specimens with random fibers is somewhere in between. These findings support the interpretation of the erosion rate of specimens with different orientation fibers, which are beneficial to analyzing the loss of particles intuitively. 

### 3.2. Macroscopic Observation of a Specimen

#### Erosion Ratio of Specimens

**Figure 11:**
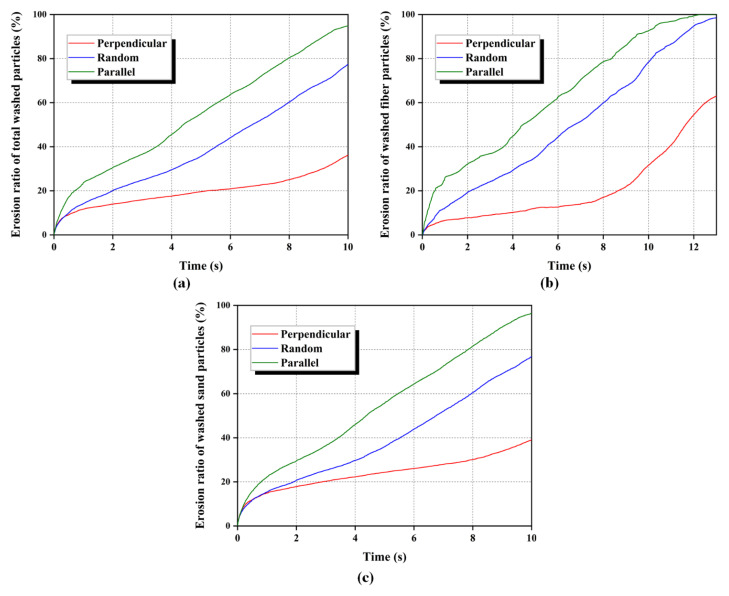
Erosion ratio of different orientated fiber-reinforced sand with time. (**a**) Total washed particles, (**b**) Fiber particles, and (**c**) Sand particles.

Figure 11 presents the variation of the erosion ratio of fiber-reinforced sands with different orientations of fibers during seepage-induced erosion. It can be seen that both fibers and sand particles in specimens with a distribution parallel to the seepage direction are washed out the fastest at a uniform hydraulic gradient. Thus, the total washed particles increase significantly in specimens with parallel fibers, followed by random fibers, and finally those with perpendicular fibers. These findings can be explained by the fact that the perpendicularly distributed fibers mobilise the largest tensile force to provide the largest resistance for sand particle flow, which limits the washed rate of sand particles. The parallelly distributed fibers mainly supply the friction at the sand-fiber interface, whose contribution to resisting the flow of sand particles is limited. Once the seepage force exceeds the frictional force, there is no restriction from the parallelly distributed fibers on the erosion of sand particles in the specimen. These explanations will be described in detail from a microscopic perspective later. This result is consistent with the experimental results of Yang, Wei, Adilehou, and Ho [14]. They found that the internal erosion resistance of fiber-reinforced soil did not improve measureably when the fibers were oriented in parallel to the seepage direction. Likewise, Devipriya et al. [40] also observed that the plastic flakes oriented perpendicular to the seepage direction play the role of a barrier for seepage flow.

### 3.3. Mesoscopic Observation

#### 3.3.1. Particle-Displacement Field of Specimens

**Figure 12:**
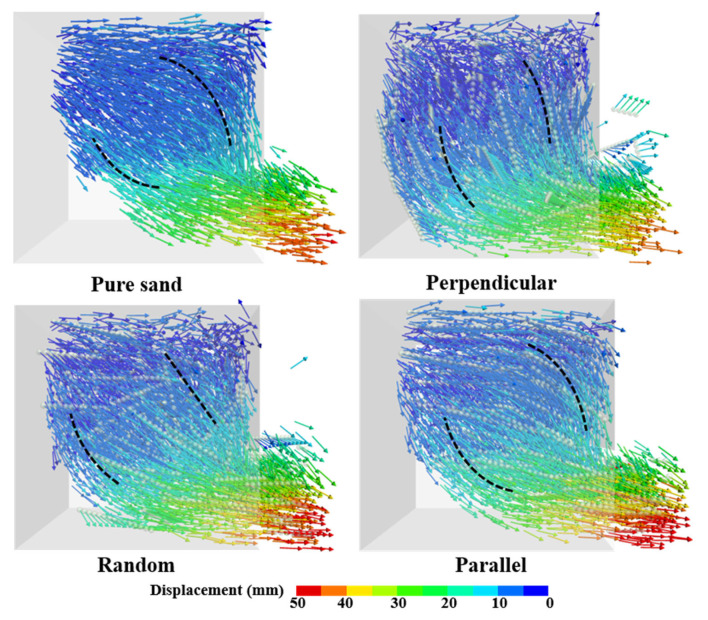
The displacement field for reinforced sand with different orientation fibers.

The visualization of the particle-displacement field for fiber-reinforced sand subjected to seepage has been instrumental in understanding the erosion resistance mechanisms of adding fibers. Figure 12 shows a typical particle-displacement field of pure sand and three fiber reinforcement sand specimens from the DEM simulation at 3 s. The direction of the arrows denotes the direction of particle movement, at that moment and the color of the arrows represents the magnitude of particle displacement since the initial state. An analysis of the displacement field is facilitated to clarify the development of erosion during the seepage process. It can be seen that the particles adjacent to the orifice at the bottom of the specimen are washed out the fastest from Figure 12, but the motion of the particles above the orifice is inconsistent. For pure sand, the particles at the top of the specimen have flowed with the streamline. For the fiber-reinforced specimen with the fibers perpendicular to the seepage direction; the obstruction of the fibers causes most of the particle to move in a direction that tends to be vertical. The path of horizontal movement above the orifice is longer for the specimen with the fibers parallel to the seepage direction, the horizontal stratification of particles motion leads to the fibers and sand particles being easier scoured. The path of displacement for randomly distributed fiber specimens shows behavior between horizontal and near-vertical. The distinction between these displacement results can explain the different rates of erosion ratios for pure sand and these three specimens. The presence of fibers impedes the movement of sand particles. However, fibers parallel to the seepage direction show noneffective resistance for the sand matrix, and the wash-out path was similar to the pure sand as well as the streamline. In conclusion, the perpendicular fibers change the washed-out path of sand particles in line with the streamline, and this behavior mitigates the rate at which the specimen is dismissed by the water flow.

#### 3.3.2. Fiber-Sand Contact Behavior

**Figure 13:**
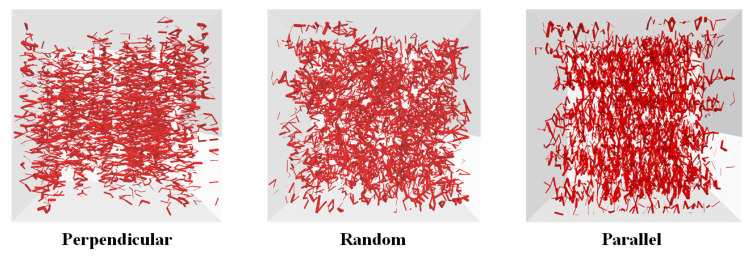
Distribution of fiber-sand contacts at the initial state (scaled by force magnitude).

In addition to sand-sand contacts, fiber-sand contacts are another contributor to the resistance of seepage forces. Especially for fibers with different orientations, the differences in the particle-scale behavior at the fiber-sand interface are important to investigate. As shown in Figure 13, the direction of fiber-sand contacts in three specimens presents a difference at the initial state. The fiber-sand contacts in the specimen with perpendicular fibers tend to be horizontally distributed, while they tend to be vertically distributed in the specimen with parallel fibers. It presents anisotropy for fiber-sand contacts in the specimen with randomly distributed fibers. As a result, the variations in micromechanical behavior at the sand-fiber interface and contacts with seepage progress are analyzed below.

##### Three-Dimensional Fabric: Evolution of Fiber-Sand Contact Type

**Figure 14:**
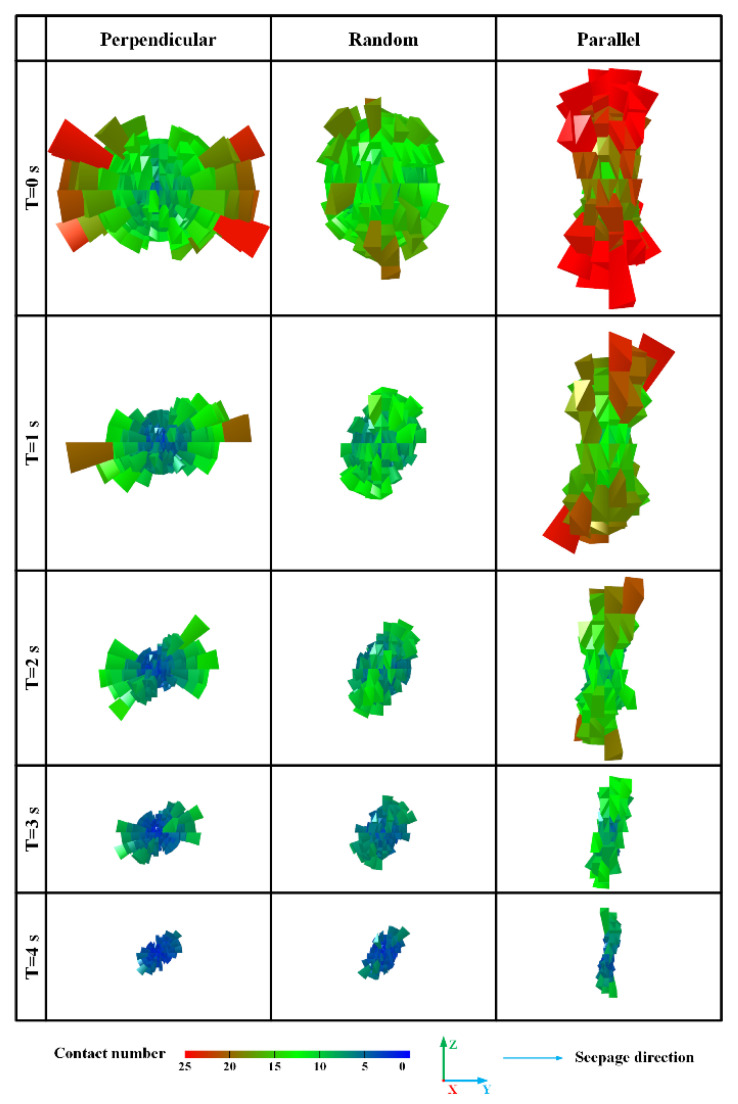
Three-dimensional fabric evolution of fiber-sand contacts for these three specimens.

The orientation evolution of contact forces can be qualitatively analyzed by counting and extracting the number of contacts in each direction. In this study, a customized code was used to export and plot the three-dimensional distribution of sand-fiber contacts. Therefore, the evolution of contact angles between the sand matrix and fibers for fiber-reinforced sand specimens with different orientations of fibers can be intuitively perceived from the three-dimensional fabric diagram. Figure 14 illustrates the three-dimensional fabric evolution of fiber-sand contacts at the moments of 0 s, 1 s, 2 s, 3 s, and 4 s (since the start of seepage). The color and length of branch vectors jointly denote the number of contacts in this direction. Note that the positive direction of seepage is horizontal to the right, as marked in Figure 14. As a whole, the number of contacts in all directions decreases with the loss of particles. It can be seen that the fiber-sand contacts are oriented mainly towards the horizontal in the initial state for perpendicular fiber specimens, while the parallel fiber specimens are mainly towards the vertical. In comparison, a randomly distributed fiber-reinforced specimen shows a slight degree of anisotropy. These indicate that the fibers perpendicular to the seepage direction can provide support to the sand matrix for resisting seepage forces at the outset. Though these distribution forms of fiber-sand contact for perpendicular and parallel fibers specimens still incline to the horizontal and vertical direction, respectively, in the following moments. The number of fiber-sand contacts is mainly concentrated between the upper and right directions, which are perpendicular to the seepage direction of randomly distributed fibers in Figure 14 (at 3 s). The reason for this is that the majority of particles have been lost, and the remaining particles are mainly concentrated in the upper right corner of the vessel. In conclusion, the diagram of the three-dimensional distribution of sand-fiber contacts can well explain the resistance of perpendicular fibers to the sand matrix in fiber-reinforced specimens at the beginning of seepage.

##### Coordination Number between Fiber-Sand Interface

There are two main mechanisms for the fibers to improve the seepage resistance of the sand matrix: the fiber netting effect and the vertical reinforcing effect [14]. The fiber netting effect is related to the friction between the fibers and sand particles. The presence of friction can significantly prevent the sand particles from being washed out, which is determined by the contact area and force of the fiber-sand interface. Besides, the scouring rate of the fibers dispersed in the sand matrix likewise decreases due to friction. The vertical reinforcing effect is provided by the potential tensile force of fibers to limit the movement of the sand matrix under seepage-induced erosion. Consequently, the effect of fibers with different orientations on improving the seepage resistance of sand particles deserves to be investigated from the perspective of micromechanics.

**Figure 15:**
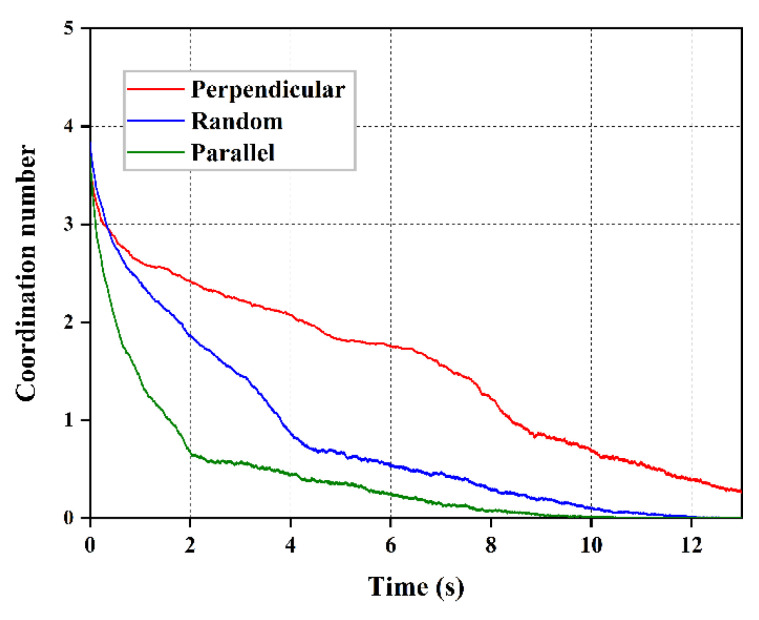
Evolution of coordination number between the fiber-sand interface.

The sparseness of specimen grains in the vessel is constantly evolving under the driving force of the seepage. The effective area of the fiber-sand interface can reflect the degree of resistance derived from the different orientations of the fibers. In this section, to quantitatively evaluate the density of the contacts between fibers and sand particles, the coordination number (CN) was adopted as a measurement parameter. The coordination number (Z*_s_*_-*f*_) of the fiber-sand interface can be calculated following Minh and Cheng [41] as:

(9) $$Z_{s-f}=\frac{2N_{c}^{s-f}}{N_{p}}$$

where the $N_{c}^{s-f}$ and $N_{p}$ is the number of fiber-sand contacts and the number of total particles in the vessel, respectively. Figure 15 shows the variation of the coordination number for the interface between different orientation fibers and the sand matrix that accompanies the seepage-induced erosion. Under the action of seepage force, the coordination number between the fiber and sand matrix interfaces gradually decreases with the loss of particles. However, the rate of this decline is distinct for the three specimens with different oriented distributions of fibers. The fibers parallel to the seepage direction are the fastest for a coordination number of 0, while the fibers perpendicular to the seepage direction are the slowest. This implies that the efficiency of fibers perpendicular to the seepage direction in supporting the sand particles to resist the seepage force is always the highest. These findings help to explain how the contact area between the sand matrix and different oriented fibers changes during seepage.

##### Mean Normal Force of Fiber-Sand Contacts

**Figure 16:**
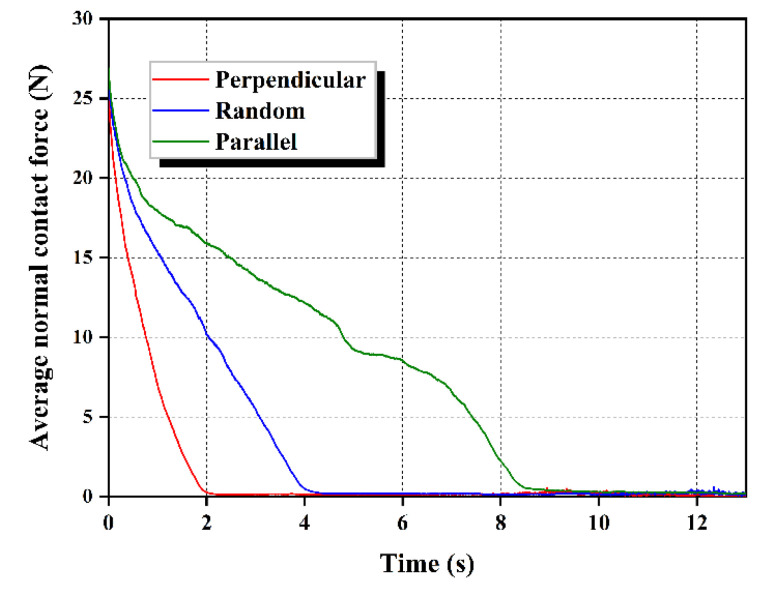
Evolution of the mean normal force acting on the fiber-sand interface.

In addition to the analysis of the coordination number between fibers and the sand matrix, the mean normal force of fiber-sand contacts has also been instrumental in clarifying the variation of internal friction. The mean normal force acting on the fiber-sand interface can reveal the influence of fiber orientation, which determines the frictional effect at the surface of the inter-particle contact. The mean normal contact force at the surface of fiber-sand is calculated as the mean value of total fiber-sand contacts. Figure 16 shows the evolution of the mean normal force acting on the surface between sand and different orientation fibers. This figure can provide a remarkable comparison for these three orientation specimens, which shows that the mean normal force is the highest during the seepage for the fibers perpendicular to the seepage direction, followed by randomly distributed fibers, and last by parallelly distributed fibers. The results illustrate that the superiority of fibers oriented perpendicular to the seepage direction can retard the decline of normal contact force on the fiber-sand surface until it disappears. As a result, the higher normal force at the point of contact between perpendicular fibers and sand particles provides higher friction to keep washed sand particles relatively stable.

#### 3.3.3. Development of Fiber Tensile Force

Yang, Wei, Adilehou, and Ho [14] have reported that fibers prevent the increase of a seepage-induced soil volume during the seepage by providing tensile resistance. In the course of seepage-induced erosion, fibers, are extruded and rubbed by the surrounding sand particles, with the result that a potential tensile force is developed. In addition, fibers mobilize their internal tensile forces to support and sustain the relative stability of the sand particles in the water flow. Thus, the development of tensile force in fibers is analyzed in this section.

**Figure 17:**
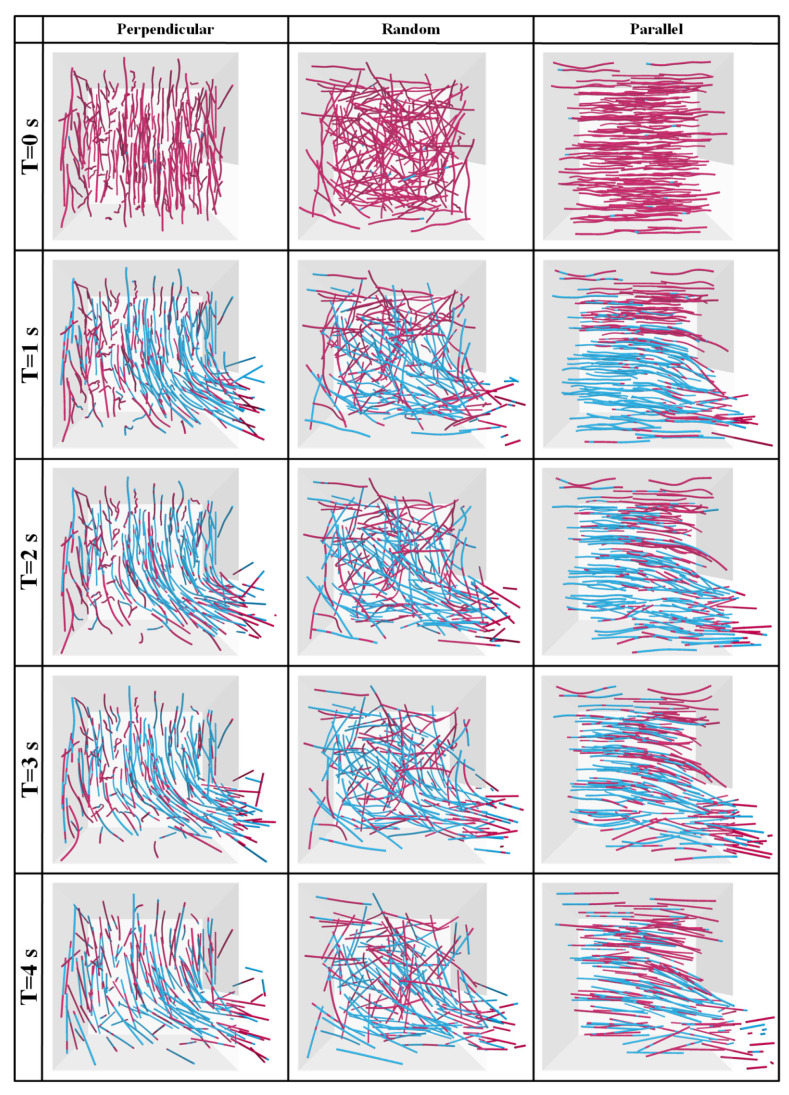
The spatial distribution of the tensile and compressive forces with time (the sand particles are not shown).

Figure 17 shows the spatial distribution of the tensile and compressive forces in different oriented fibers at the moments of 0 s, 1 s, 2 s, 3 s, and 4 s (since the start of seepage). As a whole, the tensile force of the fibers in all three specimens has increased as the seepage has progressed. For the specimen with the perpendicularly distributed fibers, the tension-forming fibers are mainly concentrated in the right half of the vessel at the beginning of seepage. This phenomenon can be explained by the fact that the sand particles near the outlet are loosened up first and moved under the action of the seepage force. To restrict the migration of sand particles in this region, the fibers rapidly mobilize the tensile resistance inside derived from the extrusion of sand particles. Tensile resistance is also developed through friction and interlocking at the fiber and matrix interface. Whereas, it can be seen that the deformation of fibers in this area is significant, which indicates that the tensile force is mainly formed by the squeeze of the surrounding matrix. The larger bending of the fibers indicates that the seepage-induced tension has exceeded the tensile resistance provided by fibers in this area. Although the presence of fibers perpendicular to the seepage direction ultimately failed to prevent the sand particles from being flushed, the migration of the sand particles is retarded to some extent.

For the specimen with the parallelly distributed fibers, the tension-forming fibers are mainly concentrated in the bottom half of the vessel at the beginning of seepage. The fibers parallel to the seepage direction cannot exert the same “netting” effect as the perpendicularly and randomly oriented fibers. The development of the tensile force within the parallel fibers is largely attributed to friction and interlocking with surrounding sand particles. However, it is finite for the effect of friction and interlocking at the fiber-sand surface. It can be seen that the tension inside the fibers is alternated with compression when the slippage occurs on the surface of fiber and sand particles, more obviously at the outlet.

For the randomly distributed fibers, the source of tensile force formation is in a transitional state between the friction and extrusion of sand particles. Therefore, the spatial distribution of the tension is spread out over randomly distributed fibers. To quantitatively describe the development of tension inside different orientation fibers, the tensile and compressive forces in fibers have been recorded and measured by the positive and negative values of force in the parallel-bond contacts. The state of parallel bond contact is defined as tension if the force is negative, and compression if the force is positive.

**Figure 18:**
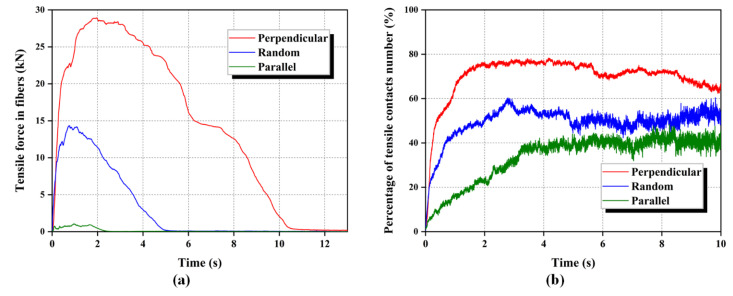
Evolution of tensile force for differently oriented fibers during the seepage: (**a**) tensile force developed in fibers, (**b**) the proportion of the number of tensile contacts in fibers.

Figure 18 illustrates the development of tensile force and the evolution of the proportion of tensile contact numbers inside different oriented fibers during the seepage. As shown in Figure 18a, fibers perpendicular to the seepage direction have the highest peak value of tension, while the lowest is for the fibers parallel to the seepage direction. This result indicates that the extrusion of sand particles can excite more tension in fibers than the friction and interlocking effects at the fiber-sand interface. Meanwhile, the proportion of the number of tensile contacts in the total contacts of fibers is the highest for the perpendicularly distributed fibers during the seepage (Figure 18b). The highest proportion of tensile force in perpendicular fibers can reach 80%, while parallel fibers are only 40%. These results explain the difference in the washed-out rate in the specimens with three orientation fibers. The tension is mobilized inconsistently in fibers with different orientations, which determines the ability of the fibers to limit the motion of surrounding sand particles; the greatest are the perpendicularly distributed fibers, and the smallest are the parallelly distributed fibers. These findings better confirm the point that the fibers exert a netting effect to restrict the flow of sand particles, as proposed by Yang, Wei, Adilehou, and Ho [14].

#### 3.3.4. Dissipation of System Energy

**Figure 19:**
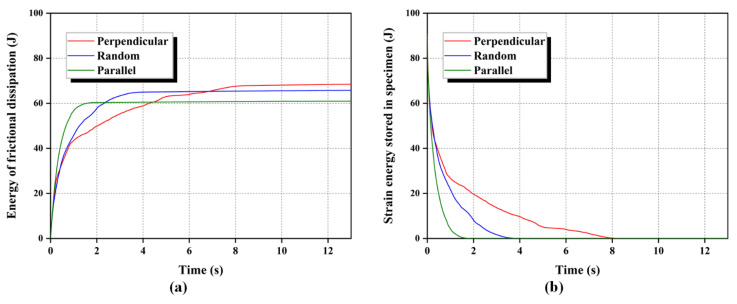
Evolution of system energy with time: (**a**) accumulated dissipation of frictional energy, (**b**) dissipated strain energy.

The energy of the DEM system is dissipated during the seepage because of the loss of particles. Hence, it is important to analyze the dissipation of energy when discussing the influence of fiber orientation. As mentioned before, the friction at the fiber-sand surface is the main reason for impeding the flow of sand particles. However, the seepage resistance relies on friction, which eventually fails under continuous seepage, and the frictional dissipation energy is consequently generated with the sliding between sand particles and fibers. Additionally, the particles within the specimen in its initial state have greater strain energy due to the mutual overlapping caused by the compaction of specimens. Subsequently, the strain energy stored in the specimen may drop owing to the loss of particles and release of overlap with the seepage. To accurately obtain the variation of system energy, the dissipation of frictional energy and the reduction of strain energy during the seepage were mainly monitored. Figure 19 shows the evolution of frictional energy and strain energy in the DEM system. For the dissipation of frictional energy, the specimen with the parallel oriented fibers has the fastest accumulation rate but the least amount of total dissipated frictional energy. Contrary to the parallel fiber specimen, the perpendicular fiber specimen show a lagging accumulation and the highest dissipation of frictional energy. Meanwhile, the dissipation rates of strain energy for these three specimens, in order from the highest to the lowest, are parallel, random, and perpendicular. These results illustrate that the fibers perpendicular to the seepage direction efficiently reduce the dissipation of system energy, which is instrumental in further identifying the effect of fiber orientation.

## 4. Conclusions

There are a lack of investigations on the contribution of fibers to the resistance of sand matrix in fiber-reinforced sands during seepage-induced erosion, especially from a mesoscopic perspective. Hence, this study conducted a series of numerical simulations of fiber-reinforced sand with different orientations of fibers under seepage-induced erosion based on the coupling method of DEM-Darcy. The macroscopic and mesoscopic behaviors of different orientation fiber specimens were respectively discussed and disclosed from simulation results. The following conclusions were obtained:

1. The presence of fibers can improve the resistance to seepage in a sand matrix, and this strengthening effect is influenced by the fiber orientation. The fibers perpendicular to the seepage direction exert the optimal hindrance effect.
2. There are differences in the permeability of specimens with different orientation fibers. Fibers perpendicular to the seepage direction increase the difficulty of moving fluid through the specimen, as embodied in the fact that the specimen with perpendicularly oriented fibers has a higher water pressure in the same location of the fluid cells. Correspondingly, the particles in the specimen with perpendicular fibers are the hardest to be washed out within these three specimens, followed by random fibers, and lastly, parallel fibers. These results can be visually recognized from the evolution of the contour graph of drag force.
3. The particle-displacement field is different for the specimens with different orientation fibers. With the addition of fibers, the movement of sand particles is changed and no longer follows the streamline. The flow path of sand particles is strongly warped in the perpendicularly oriented fibers, and this phenomenon explains why the washed-out rate of particles is reduced mostly in that specimen.
4. Compared with randomly and parallelly distributed fibers, the presence of fibers perpendicular to the seepage direction can provide contact support between the sand and fiber interface in the initial state. The average normal force and contact area of this contact used to resist seepage force in perpendicularly oriented fiber specimens are always higher than those of randomly and parallelly oriented fiber specimens until the sand-fiber contacts are completely lost. These behaviors have been confirmed by monitoring the evolution of energy dissipation.
5. (During the seepage-induced erosion, the fibers perpendicular to the seepage direction can mobilize more tensile force to limit the motion of surrounding sand particles. These tensile forces mainly derive from the extrusion of surrounding sand particles. In contrast, the tensile forces developed in parallel fibers are the smallest, which is driven by the friction between the fibers and the sand surface. According to the simulation results, it is found that the highest proportion of tensile force in perpendicular fibers can reach 80%, while parallel fibers are at 40%. These results have a better explanation for the point in the study of Yang, Wei, Adilehou and, Ho [14], that is, the fiber netting effect. The findings of this paper can be beneficial to further understanding the contribution of fibers to resisting the wash of sand matrix.

## Figures and Tables

**Table 1:** Contacts model selection of DEM model.

Contact Types	Contact Models
Sand-sand	Rolling resistance linear model
Sand-fiber	Linear model
Fiber-fiber	Linear model
Within fibers	Linear parallel bond model

**Table 2:** Microscopic non-bonding parameters calibrated in this study.

Parameters	Value			
	Contact Type			
	Wall-Ball Contacts	Sand-Sand Contacts	Sand-Fiber and Fiber-Fiber (External) Contacts	Fiber-Fiber Contacts (Internal)
Effective modulus/Pa	1.5 × 10^8^	1.5 × 10^8^	1.8 × 10^8^	2.6 × 10^8^
Normal to shear stiffness ratio	1	1	1	2
Friction coefficient	0	0.3	0.5	0.8
Rolling resistance coefficient	N/A	0.32	N/A	N/A

**Table 3:** Bonding parameters for fiber-fiber contacts (internal).

Parameters	Parallel Bond Effective Modulus/Pa	Normal Parallel Bond Shears Stiffness Ratio	Tensile Strength/Pa	Cohesion/Pa
Value	1 × 10^9^	2	1× 10^12^	1 × 10^12^

Note that the bonding parameters shown in Table 2 are only assigned to the particles inside individual fibers. There is no bonding between two separate fibers.

## Data Availability

Data or code will be made available on request.
